# Incidence of COVID-19-Associated Hospitalization by Vaccination Status in Children and Adolescents During Omicron-Dominant Period in Japan: The VENUS Study

**DOI:** 10.3390/children13020183

**Published:** 2026-01-28

**Authors:** Haruka Maeda, Michiko Toizumi, Ataru Igarashi, Megumi Maeda, Futoshi Oda, Haruhisa Fukuda

**Affiliations:** 1 Department of Respiratory Infections, Institute of Tropical Medicine, Nagasaki University, Nagasaki 852-8523, Japan; 2 Department of Pediatric Infectious Diseases, Institute of Tropical Medicine, Nagasaki University, Nagasaki 852-8523, Japan; 3 Department of Health Policy and Public Health, Graduate School of Pharmaceutical Sciences, The University of Tokyo, Tokyo 113-0033, Japan; 4 Department of Health Care Administration and Management, Kyushu University Graduate School of Medical Sciences, Fukuoka 812-8582, Japan

**Keywords:** COVID-19, SARS-CoV-2, vaccination, children, adolescents, hospitalization, Omicron, Japan

## Abstract

**Highlights:**

**What are the main findings?**

- Receipt of at least two doses of an ancestral monovalent COVID-19 vaccine was associated with a lower incidence rate of COVID-19-associated hospitalization among children and adolescents (adjusted IRR 0.429; 95% CI: 0.198–0.930).
- Only 4.3% of COVID-19-associated hospitalizations required oxygen administration, whereas 36.6% involved diagnoses related to vomiting, diarrhea, hypovolemia, or hypoglycemia, indicating that gastrointestinal symptoms and dehydration were common reasons for hospitalization.

**What are the implications of the main findings?**

- The results provide real-world evidence that pediatric COVID-19 vaccination was associated with a lower incidence rate of COVID-19-associated hospitalization in Japan during the Omicron-dominant period.
- Expanding COVID-19 vaccine uptake among eligible pediatric populations may reduce the future burden of severe disease.

**Abstract:**

**Background/Objectives**: Evidence regarding the impact of COVID-19 vaccination on children and adolescents remains limited, particularly in Japan. Thus, this study aimed to estimate incidence rates of COVID-19-associated hospitalization by vaccination status and evaluate the association between COVID-19 vaccination and COVID-19-associated hospitalization among individuals aged 6 months to <18 years in Japan. **Methods**: We conducted a retrospective, population-based cohort study using linked health insurance claims data and municipal COVID-19 vaccination registry records from nine Japanese municipalities between 1 January 2022, and 31 March 2023, when the Omicron variant was dominant nationwide. Incidence rates of COVID-19-associated hospitalization were estimated among unvaccinated individuals and those who had received one dose (partially vaccinated) or at least two doses of an ancestral monovalent COVID-19 vaccine (fully vaccinated). Incidence rate ratios (IRRs) were calculated, adjusting for age group, sex, presence of underlying medical conditions, municipality, and calendar month. **Results**: Among 163,305 individuals, 93 COVID-19-associated hospitalizations were identified. Crude incidence rates were 4.5 (95% confidence interval [CI]: 3.6–5.6), 3.2 (95% CI: 0.1–18.0), and 2.3 (95% CI: 1.1–4.2) per 100,000 person-months in unvaccinated, partially, and fully vaccinated groups, respectively. The adjusted IRR for the fully vaccinated group was 0.429 (95% CI: 0.198–0.930). Among hospitalized patients, 30.1% had underlying medical conditions, and only 4.3% required oxygen administration during hospitalization. **Conclusions**: Receiving at least two doses of COVID-19 vaccine was associated with a lower incidence rate of COVID-19-associated hospitalization among children and adolescents during the Omicron-dominant period in Japan. Expanding vaccine uptake among eligible pediatric populations may help reduce the burden of severe disease.

## 1. Introduction

The severe acute respiratory syndrome coronavirus 2 (SARS-CoV-2) Omicron variant began spreading in Japan at the end of 2021 and rapidly became the predominant circulating variant [1]. Although the risk of severe disease in children and adolescents was lower than in older adults [2], Omicron variant was reported to be highly transmissible and spread widely among younger populations [3,4]. This surge was accompanied by an increased number of pediatric hospitalizations and severe cases compared with those caused by previous variants [5,6,7,8]. Studies from the United States and Canada reported that hospitalization rates among children increased during the Omicron-dominant compared with the Delta-dominant period [7,8].

In this context, coronavirus disease 2019 (COVID-19) vaccination became increasingly important for preventing severe outcomes in children and adolescents. However, vaccine protection against COVID-19-associated hospitalization was reported to be lower during the Omicron-dominant than the Delta-dominant period among adolescents. For example, a test-negative study from the United States reported that vaccine effectiveness against hospitalization among adolescents aged 12–18 years decreased from 93% during the Delta-dominant period to 40% during the Omicron-dominant period, although protection against critical illness remained marked [9]. Nevertheless, multiple studies demonstrated that COVID-19 vaccination continued to provide protection against hospitalization during the Omicron-dominant period among children, with vaccine effectiveness of 68% in the United States [9], 82.7% in Singapore [10], and 41.1% against severe COVID-19 in Italy [11] among children aged 5–11 years.

In Japan, COVID-19 vaccines were introduced for children and adolescents in a stepwise manner: in June 2021 for individuals aged ≥12 years, in February 2022 for those aged ≥5 years, and in October 2022 for those aged ≥6 months [12,13,14]. An epidemiological study of pediatric hospitalized COVID-19 patients in Japan reported an increase in pediatric hospitalizations during the Omicron period [12]. Importantly, the proportion of vaccinated children among hospitalized patients was lower than that in the general population (11.1 vs. 41.5% among children aged 5–11 years; 38.9 vs. 71.0% among those aged 12–15 years, respectively) [15], suggesting a potential protective effect of COVID-19 vaccination. However, despite recommendations from the Japan Pediatric Society [16,17], vaccine coverage in these age groups has remained low [13].

Understanding whether COVID-19 vaccines are effective for children and adolescents is important for informing public health policy as well as promoting individual and family decisions regarding vaccination. However, epidemiological evidence from Japan remains limited, particularly population-based studies estimating incidence rates and evaluating the association between vaccination and COVID-19-associated hospitalization during the Omicron-dominant period. Therefore, this study aimed to estimate incidence rates of COVID-19-associated hospitalization by COVID-19 vaccination status and describe the characteristics of hospitalized COVID-19 patients (children and adolescents) in Japan during the Omicron-dominant period. In addition, we calculated the incidence rate ratio (IRR) for the outcome in vaccinated versus unvaccinated populations to assess the association between COVID-19 vaccination and the risk of COVID-19-associated hospitalization within this age group.

## 2. Materials and Methods

### 2.1. COVID-19 Vaccination Strategy for Children and Adolescents in Japan

In Japan, BNT162b2 was approved on 14 February 2021 for individuals aged ≥16 years, and the COVID-19 vaccination campaign started in February 2021, initially targeting healthcare workers, then older adults in April 2021, and it expanded to include younger populations [18]. BNT162b2 was authorized for ages 12–15 in May 2021, and its nationwide administration to individuals aged 12–15 years subsequently commenced [13]. For individuals aged 5–11, BNT162b2 received approval on 21 January 2022, with implementation in late February 2022 [13]. BNT162b2 was then approved for ages 6 months to <5 years in October 2022 [19], and vaccination began for this group in late October. In parallel, additional types of COVID-19 vaccines became available for adolescents: mRNA-1273 was approved for ages 12–17 in July 2021 [20], and then NVX-CoV2373 for ages 12–17 in July 2022 [21].

COVID-19 vaccination for individuals aged ≥6 months had been publicly funded up until 31 March 2024. As of March 2024, approximately 80% of eligible individuals and 95% of older adults aged ≥65 years had received at least two doses. In contrast, vaccination coverage was markedly lower among children and infants: 24% among those aged 5 to <12 years, and 4% among those aged 6 months to <5 years [22].

### 2.2. Study Design, Population and Data Source

We conducted a retrospective, population-based cohort study from 1 January 2022, to 31 March 2023, when the Omicron variant was predominant nationwide in Japan. We used linked health insurance claims data and municipal COVID-19 vaccination registry records from nine Japanese municipalities. This study was a part of Vaccine Effectiveness, Networking, and Universal Safety (VENUS) Study [23].

We included children and adolescents aged 6 months to <18 years who were enrolled in municipality-funded health insurance programs (Supplementary Table S1) and resided in one of the nine municipalities. For individuals aged ≥1 year at the start of the study period (1 January 2022), continuous registration in the health insurance system was required to ensure adequate inclusion in the health insurance system during the study period and to assess underlying medical conditions. Specifically, individuals were required to have at least one claim recorded by December 2020 and at least one claim during the 12 months before 1 January 2022. For infants aged <1 year on 1 January 2022, and for those born after 1 January 2022, at least one claim between birth and the cohort entry date (CED) was required. Because the cohort was based on municipality-funded health insurance claims, individuals with no recorded healthcare utilization during the specified period were excluded, irrespective of COVID-19 vaccination status. We excluded individuals who had moved out of the municipality or died before CED, as well as those with missing COVID-19 vaccination history.

Since this study aimed to estimate incidence rates of COVID-19-associated hospitalization during the Omicron epidemic period among children and adolescents aged 6 months to <18 years, CED was defined as 1 January 2022 [24], or the date the child reached 6 months of age, whichever came later. Participants were followed from CED until the earliest of the following: (1) occurrence of the outcome event, (2) relocation outside the municipality, (3) their 18th birthday, or (4) the end of the study period (31 March 2023). The study period was chosen to correspond to the period when Omicron was dominant in Japan and COVID-19 was classified as a Category II infectious disease, during which SARS-CoV-2 testing and treatment were fully subsidized by public funding.

### 2.3. Outcomes, Exposure, and Covariates

The primary outcome was COVID-19-associated hospitalization, defined as any hospital admission with both: (1) an International Classification of Diseases, Tenth Revision (ICD-10) diagnosis codes of U07.1 (COVID-19), B34.2 (coronavirus infection, unspecified), or U10.9 (multisystem inflammatory syndrome in children: MIS-C), and (2) a procedure code indicating COVID-19-associated hospitalization (Supplementary Table S2) [25]. This definition improves the positive predictive value for detecting COVID-19-associated hospitalizations compared with the use of ICD-10 codes alone in Japan [25]. Because several hospitalizations may have occurred primarily for reasons other than COVID-19 with incidental SARS-CoV-2 detection, we defined a secondary outcome as COVID-19-associated hospitalizations excluding hospitalizations with trauma-related diagnoses (ICD-10 codes S00–S99) or surgical procedure codes [26].

The exposure of interest was receipt of one dose or at least two doses of an ancestral monovalent COVID-19 vaccine, with the exposure status defined as from ≥14 days after the receipt of the second dose. Covariates included age group (6 months to <5 years, 5 to <12 years, and 12 to <18 years), sex, municipality of residence, calendar month and presence of underlying medical condition, defined by pediatric complex chronic conditions (Supplementary Table S3) [27]. Underlying medical conditions were evaluated over the 12 months before CED for individuals aged 1 to <18 years at CED, and from birth to CED for those aged 6 months to <1 year at CED.

### 2.4. Statistical Analysis

Vaccination status was categorized into three groups: (1) unvaccinated, (2) partially vaccinated (defined as receiving one dose of an ancestral monovalent COVID-19 vaccine until <14 days after the second dose), and (3) fully vaccinated (defined as receiving at least two doses, with ≥14 days having elapsed since the second dose). Follow-up time was divided into three periods. For initially unvaccinated individuals, the unvaccinated period extended from CED until receipt of the first COVID-19 vaccine dose, and the partially vaccinated period began from the date of receiving the first dose. Then, the fully vaccinated period began 14 days after receipt of the second dose. Individuals who had received only one dose before CED contributed person-time to the partially vaccinated group from CED until 14 days after receipt of the second dose if they received the second dose. Individuals who had already received at least two doses before CED contributed person-time to the fully vaccinated group.

Categorical variables are summarized as frequencies and proportions, and continuous variables as medians and interquartile ranges (IQRs). When comparing two proportions, the Chi-squared test was used. Crude incidence rates of COVID-19-associated hospitalization were calculated as the number of events per 100,000 person-months for each vaccination category. Exact 95% confidence intervals (CIs) were obtained from the Poisson distribution. For strata with zero events, the lower confidence limit was set to zero. Characteristics and clinical outcomes of hospitalized patients were also described. Incidence rates were calculated overall and stratified by age group, presence of underlying medical condition, and periods of dominant Omicron subvariants, defined based on national variant surveillance (BA.1/BA2, January–June 2022, BA.5, July–November 2022, and BA5/BQ.1, December 2022–March 2023) [24]. IRR with 95% CIs for partially vaccinated or fully vaccinated versus unvaccinated participants was estimated using Poisson regression models with a log(person-time) offset. Models were adjusted for age group, sex, municipality of residence, presence of underlying medical condition and calendar month. Because some individuals could contribute person-time to more than one vaccination group, we used cluster-robust standard errors with clustering at the individual level to account for the within-individual correlation. A negative binomial regression model was also fitted to assess overdispersion.

Individuals were categorized into three groups based on age: 6 months to <5 years, 5 to <12 years, and 12 to <18 years. When aging inevitably led to progression to a higher category during follow-up, their person-time was reassigned to the new age group from that date onward. A two-sided *p*-value of <0.05 was considered significant. All analyses were performed using Stata version 17 (StataCorp LLC, College Station, TX, USA).

### 2.5. Ethics

This study was approved by the Kyushu University Institutional Review Board for Clinical Research (no.: 22114-00). The requirement for obtaining informed consent from individual participants was waived in accordance with the Ethical Guidelines for Medical Research Involving Human Subjects in Japan.

## 3. Results

### 3.1. Characteristics of the Study Population

**Figure 1:**
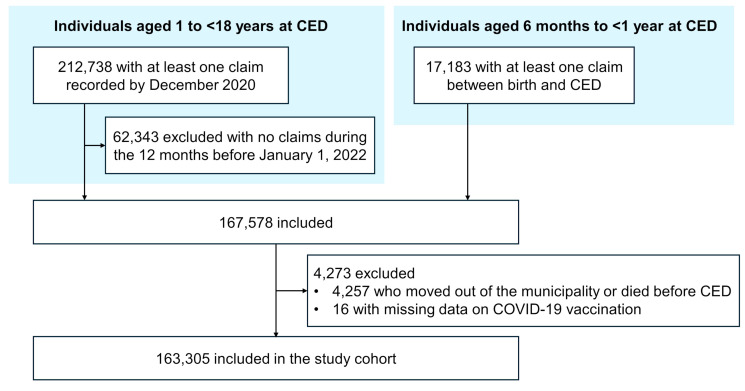
Flow chart of the study.

Among 229,921 individuals aged 6 months to <18 years from the nine municipalities, 163,305 were included in the analysis (Figure 1). Overall, 51,977 (31.8%) were aged 6 months to <5 years, 75,650 (46.3%) 5 to <12 years, 79,182 (48.5%) were female, 11,348 (6.9%) had at least one underlying medical condition, and 5010 (3.1%) had a history of hospitalization within six months before CED (Table 1). At CED, 142,681 (87.4%) were unvaccinated and 20,015 (12.3%) had received at least two COVID-19 vaccine doses. Demographics and characteristics by vaccination status as of 31 March 2023, are summarized in Table 2. By the end of the study period, 39,207 (24.0%) had received at least one dose. The proportion of vaccinated individuals differed by age group: 3.3% among infants and children aged 6 months to <5 years, 19.0% among those aged 5 to <12 years, and 64.8% among adolescents aged 12 to <18 years.

**Table 1:** Characteristics of children and adolescents aged 6 months to <18 years in the study cohort, 1 January 2022, to 31 March 2023, Japan.

	Total, no. (%) (n = 163,305)
Age group (at CED ^a^)	
6 months to <5 years	51,977 (31.8)
5 to <12 years	75,650 (46.3)
12 to <18 years	35,678 (21.8)
Female sex, no. (%)	79,182 (48.5)
Presence of underlying medical condition, no. (%)	
Any	11,348 (6.9)
Neurologic	2446 (1.5)
Cardiovascular	2023 (1.2)
Respiratory	282 (0.2)
Renal and urologic	764 (0.5)
Gastrointestinal	605 (0.4)
Hematologic or immunologic	1191 (0.7)
Metabolic	1508 (0.9)
Other congenital or genetic defect	2637 (1.6)
Malignancy	1228 (0.8)
Premature and neonatal	2268 (1.4)
Technology dependence	151 (0.1)
Transplantation	8 (0.005)
Number of underlying medical conditions, no. (%)	
0	151,957 (93.1)
1 to 2	10,593 (6.5)
≥3	755 (0.5)
History of hospitalization within six months before CED	5010 (3.1)
COVID-19 vaccination at CED, no. (%)	
Unvaccinated	142,681 (87.4)
One dose	609 (0.4)
At least two doses	20,015 (12.3)
COVID-19 vaccination on 31 March 2023, no. (%)	
Unvaccinated	124,098 (76.0)
One dose	961 (0.6)
At least two doses	38,246 (23.4)

Abbreviations; CED, cohort entry date; COVID-19, coronavirus disease 2019. ^a^: The cohort entry date (CED) was set at either of: (1) 1 January 2022, for those born before June 2021; (2) on reaching six months of age for those born after July 2021.

**Table 2:** Characteristics of children and adolescents aged 6 months to <18 years in the study cohort by vaccination status at the end of the study period (31 March 2023).

	Total, no.	Unvaccinated, no. (% ^a^)	Vaccinated, no. (% ^a^)
Overall	163,305	124,098 (76.0)	39,207 (24.0)
Age group (at CED ^b^)			
6 months to <5 years	51,977	50,283 (96.7)	1694 (3.3)
5 to <12 years	75,650	61,260 (81.0)	14,390 (19.0)
12 to <18 years	35,678	12,555 (35.2)	23,123 (64.8)
Sex			
Male	84,123	64,087 (76.2)	20,036 (23.8)
Female	79,182	60,011 (75.8)	19,171 (24.2)
Underlying medical condition			
Without any medical condition	151,957	115,906 (76.3)	36,051 (23.7)
With any medical condition	11,348	8192 (72.2)	3156 (27.8)
Neurologic	2446	1528 (62.5)	918 (37.5)
Cardiovascular	2023	1410 (69.7)	613 (30.3)
Respiratory	282	194 (68.8)	88 (31.2)
Renal and urologic	764	564 (73.8)	200 (26.2)
Gastrointestinal	605	391 (64.6)	214 (35.4)
Hematologic or immunologic	1191	929 (78.0)	262 (22.0)
Metabolic	1508	986 (65.4)	522 (34.6)
Other congenital or genetic defect	2637	1745 (66.2)	892 (33.8)
Malignancy	1228	800 (65.1)	428 (34.9)
Premature and neonatal	2268	2043 (90.1)	225 (9.9)
Technology dependence	151	79 (52.3)	72 (47.7)
Transplantation	8	4 (50.0)	4 (50.0)
Number of underlying medical conditions			
1 to 2	10,593	7704 (72.7)	2889 (27.3)
≥3	755	488 (64.6)	267 (35.4)
History of hospitalization ^c^	5010	4334 (86.5)	676 (13.5)

Abbreviations; CED, cohort entry date; COVID-19, coronavirus disease 2019. ^a^: Percentages are row percentages. ^b^: The cohort entry date (CED) was set at either of: (1) 1 January 2022, for those born before June 2021; (2) on reaching six months of age for those born after July 2021. ^c^: History of hospitalization within six months before CED.

The cohort entry date (CED) was defined as 1 January 2022, or the date the child reached 6 months of age, whichever came later.

### 3.2. Incidence Rates of COVID-19-Associated Hospitalization

During the study period, 93 COVID-19-associated hospitalizations were identified among individuals aged 6 months to <18 years, including 82 unvaccinated, one partially vaccinated and 10 fully vaccinated individuals. Among the 10 fully vaccinated hospitalized patients, the median interval between the most recent vaccination and date of admission was 137.5 days (range: 72–476). No cases of MIS-C were observed. For the secondary outcome, 87 COVID-19-associated hospitalizations were identified after excluding hospitalizations with trauma-related diagnoses or surgical procedure codes, including 78 unvaccinated and nine fully vaccinated individuals. In total, six hospitalizations were excluded (three fractures, one head injury, one neck injury, and one admission requiring abdominal surgery). Table 3 shows the characteristics and clinical outcomes of 93 COVID-19-associated hospitalized patients (primary outcome). The median age of such patients was 8 years (IQR: 3–12); 46 (49.5%) were female, and 28 (30.1%) had at least one underlying medical condition, a larger proportion than among non-hospitalized individuals (6.9%, *p* < 0.001). The most common underlying medical conditions were neurologic (11.8%), metabolic (8.6%), cardiovascular (7.5%), premature and neonatal (7.5%), and malignancy (4.3%). During hospitalization, four patients (4.3%) required oxygen administration, and two (2.2%) needed intubation and mechanical ventilation. Neither of the two patients who required mechanical ventilation had received any dose of COVID-19 vaccine. Among all hospitalized patients, diagnoses were related to: vomiting and diarrhea, 21 (22.6%); hypovolemia, 20 (21.5%); hypoglycemia, five (5.4%); seizure, nine (9.7%). No deaths occurred during hospitalization. The characteristics and clinical outcomes of hospitalizations meeting the secondary outcome were similar to those observed for the primary outcome (Supplementary Table S4).

Crude incidence rates of COVID-19-associated hospitalization were 4.5 (95% CI: 3.6–5.6), 3.2 (95% CI: 0.1–18.0) and 2.3 (95% CI: 1.1–4.2) per 100,000 person-months in the unvaccinated, partially and fully vaccinated (≥2 doses) groups, respectively (Table 4). IRRs for COVID-19-associated hospitalization in the partially and fully vaccinated compared with unvaccinated group were 0.975 (95% CI: 0.138–6.89) and 0.429 (95% CI: 0.198–0.930), respectively. By age group, among individuals aged 6 months to <5 years, crude incidence rates were 6.0 (95% CI: 4.2–8.4) and 0 (95% CI: 0–188.8) per 100,000 person-months in the unvaccinated and fully vaccinated groups, respectively (Supplementary Table S5). Among those aged 5 to <12 years, the corresponding rates were 3.3 (95% CI: 2.2–4.6) and 0.9 (95% CI: 0.02–5.3), and among those aged 12 to <18 years, 6.1 (95% CI: 3.4–10.0) and 2.8 (95% CI: 1.3–5.2), respectively. Among individuals with underlying medical conditions, crude incidence rates were 23.8 (95% CI: 15.8–34.4) and 0 (95% CI: 0–10.5) per 100,000 person-months in the unvaccinated and fully vaccinated groups, respectively. Incidence rates stratified by the period of dominant Omicron subvariants were as follows: during the BA.1 and BA.2-dominant period (January–June 2022), incidence rates were 5.7 (95% CI: 4.1–7.6) and 2.9 (95% CI: 0.8–7.4) per 100,000 person-months in unvaccinated and fully vaccinated groups, respectively; the corresponding rates were 5.0 (95% CI: 3.4–7.2) and 2.5 (95% CI: 0.7–6.3) during the BA.5-dominant period (July–November 2022); and 2.1 (95% CI: 1.0–4.0) and 1.5 (95% CI: 0.2–5.4) during the BA.5 and BQ.1-dominant period (December 2022–March 2023). The estimated dispersion parameter (α) from the negative binomial model was close to zero (2.1 $\times \;10^{-16}$), and the likelihood ratio test for α was not significant, which did not indicate overdispersion.

For the secondary outcome, crude incidence rates of COVID-19-associated hospitalization excluding hospitalizations with trauma-related diagnoses or surgical procedure codes were 4.3 (95% CI: 3.4–5.4) and 2.1 (95% CI: 0.9–3.9) per 100,000 person-months in the unvaccinated and fully vaccinated groups, respectively (Table 4). IRR for the fully vaccinated compared with unvaccinated group was 0.377 (95% CI: 0.174–0.816). The results of subgroup analyses are shown in Supplementary Table S6.

## 4. Discussion

In this cohort study involving a period during the Omicron epidemic, incidence rates of COVID-19-associated hospitalization were lower among individuals who had received at least two doses of COVID-19 vaccine compared with unvaccinated individuals, corresponding to IRR of 0.429 (95% CI: 0.198–0.930). Among the 93 hospitalized patients, no deaths or cases of MIS-C were identified. Overall, only 4.3% of hospitalized patients required oxygen administration, whereas 36.6% had diagnoses related to: vomiting, diarrhea, hypovolemia, or hypoglycemia, indicating that gastrointestinal symptoms and dehydration were common reasons for hospitalization in the population during this period. Our study adds population-based real-world evidence from Japan by linking vaccination registry data with insurance claims to estimate the incidence rate of COVID-19-associated hospitalization by vaccination status and association between COVID-19 vaccination and the risk of COVID-19-associated hospitalization among children and adolescents.

In our study conducted during the Omicron-dominant period, receipt of at least two doses of an ancestral monovalent COVID-19 vaccine was associated with a lower risk of COVID-19-associated hospitalization among children and adolescents aged 6 months to <18 years. These findings indicate that COVID-19 vaccination could provide protection against hospitalization in pediatric populations, even in the context of immune-evasive Omicron variants. Although our study was not designed to directly estimate vaccine effectiveness, making direct comparison with previous vaccine effectiveness studies challenging, the direction of our findings is generally consistent with previous evidence from Japan and other countries. A previous Japanese study reported moderate effectiveness of COVID-19 vaccine against severe COVID-19, defined as hospitalization with respiratory failure or death, among children aged 5–17 years during the Omicron BA.5 period, with vaccine effectiveness estimates ranging from 49 to 73% depending on the number of doses and types of vaccine [19]. In the United States, both a test-negative study and population-based surveillance reported vaccine effectiveness among children and adolescents [9,28]. A test-negative study conducted by the Overcoming COVID-19 Investigators from Centers for Disease Control and Prevention (CDC) in the United States reported that, during the Omicron-dominant period, vaccine effectiveness of BNT162b2 against COVID-19-associated hospitalization was 40% (95% CI: 9–60) among adolescents aged 12–18 years and 67% (95% CI: 42–82) among children aged 5–11 years, while vaccine effectiveness against critical COVID-19 among adolescents reached 79% (95% CI: 51–91) [9]. Population-based surveillance conducted by the New Vaccine Surveillance Network from US CDC demonstrated vaccine effectiveness of 40% (95% CI: 8–60%) against COVID-19-associated emergency department visits and hospitalization among infants and children aged 6 months to 4 years [28]. Similarly, studies from Canada reported lower risks of hospitalization among vaccinated children, including an 85% reduction among children and adolescents aged 4–17 years [29] and vaccine effectiveness ranging from 63 to 93% among children aged 5–11 years [30]. Evidence from other countries also supports vaccine effectiveness against hospitalization. A study from Singapore demonstrated marked protection (VE: 75% [95% CI: 56–86]) against Omicron-associated hospitalization among adolescents aged 12–17 years [31], and a study conducted in six European countries reported moderate protection (VE: 41–56%) against hospitalization due to COVID-19 during the Omicron BA.1/BA.2/BA.4/BA.5 periods among children and adolescents aged 5–17 years [32]. Direct comparisons across studies are challenging because of differences in study design, age groups, background infection-induced immunity at the population level, time since vaccination, outcome definitions, and vaccination schedules. Moreover, waning of protection over time has been reported even for severe outcomes [9,32,33], and our inability to stratify analyses by time since vaccination limited further direct comparison. In addition, our study was not able to evaluate dose-specific effects due to the limited sample size of hospitalized cases. Nevertheless, our study provides important real-world evidence from Japan, demonstrating that receipt of at least two doses of COVID-19 vaccine was associated with a reduced risk of COVID-19-associated hospitalization among children and adolescents during the Omicron-dominant period. These findings contribute evidence from Asia, where data on the association between COVID-19 vaccination and severe pediatric outcomes have remained limited.

The proportion of individuals with underlying medical conditions was larger among hospitalized than non-hospitalized participants (30.1 vs. 6.9%, respectively, *p* < 0.001), which is consistent with previous studies [30,34]. Neurologic disorders were the most common underlying medical conditions (11.8%), followed by metabolic (8.6%), cardiovascular (7.5%), premature and neonatal (7.5%), and malignancy (4.3%). Overall, the distribution of common underlying medical conditions observed in our study was generally similar to that reported in previous studies [15,35,36]. Notably, no COVID-19-associated hospitalizations were observed among children and adolescents with underlying medical conditions who had received COVID-19 vaccines (incidence rate: 0 [95% CI: 0–10.5] per 100,000 person-months), although sample size was limited. In contrast, the corresponding incidence rate among unvaccinated children and adolescents with underlying medical conditions was 23.8 (95% CI: 15.8–34.4) per 100,000 person-months. These findings suggest that COVID-19 vaccination may be beneficial in this high-risk group.

Our findings suggest that the reasons for hospitalization due to COVID-19 differed between children and adults during the Omicron-dominant period. In our study, only four patients (4.3%) required oxygen administration and two needed intubation and mechanical ventilation, whereas 36.6% of hospitalized children and adolescents had diagnoses related to dehydration or poor oral intake and gastrointestinal symptoms such as vomiting, and 9.7% had diagnoses related to seizures. This pattern is consistent with pediatric reports from Japan and South Africa during the Omicron-dominant period [12,15,37]. Studies from Japan reported that dehydration or insufficient oral intake accounted for 52.3% of pediatric hospitalizations during the Omicron BA.1/BA.2-dominant period [12] and approximately 40% during the Omicron BA.5-dominant period [12,15]. Seizures accounted for 15–20% of pediatric hospitalizations during these periods [12,15]. An observational study from South Africa described the clinical symptoms of hospitalized pediatric patients aged <20 years with positive SARS-CoV-2 test results during the Omicron-dominant period, reporting the proportion of symptoms as follows: fever (46%), cough (40%), vomiting (24%), diarrhea (22%), and seizure (20%) [37]. In contrast, several studies involving adults and older populations during the Omicron period reported that respiratory complications, including pneumonia, hypoxia, and respiratory failure, remain the predominant reasons for hospitalization [38,39,40,41]. These findings indicate that the clinical impact of COVID-19 may differ between children and adults, and that supportive-care needs, rather than respiratory failure, were the major drivers of hospitalization among children.

This study had several limitations. First, we included only individuals covered by municipality-funded health insurance programs, such as National Health Insurance and Public Assistance, and did not include those covered by the employment-based health insurance system. Since the insurance type in Japan is closely associated with parental employment and the socioeconomic status, our findings may not be fully generalizable to the entire pediatric population. Second, COVID-19-associated hospitalizations were identified based on the presence of both an ICD-10 diagnosis code and a procedure code related to COVID-19 hospitalization. This definition may have inadvertently included hospitalizations in which COVID-19 was not the primary reason for admission, such as for trauma or surgery in patients who were incidentally found to have COVID-19. To address this concern, we defined a secondary outcome as COVID-19-associated hospitalization excluding hospitalizations with trauma-related diagnose codes or surgical procedure codes. The results were similar to those regarding the primary outcome. Nevertheless, some misclassification and overestimation of incidence rates may have occurred. Third, due to the limited sample size, we were unable to evaluate dose-specific vaccine effectiveness or the waning of vaccine-induced immunity over time. This made it difficult to directly compare our results with those from studies that assessed detailed dose- and time-dependent effects. Fourth, this study was based on Japanese administrative insurance claims data, which were originally collected for reimbursement rather than research purposes. As a result, detailed clinical information, including disease severity, laboratory findings, and clinical decision-making leading to hospitalization, was not available. Although claims-based data are useful for large-scale epidemiologic analyses, important clinical information could not be fully accounted for, and residual confounding could not be excluded. Fifth, residual confounding due to indication and healthy vaccinee bias cannot be excluded [42,43]. In our cohort, 35.4% of individuals with ≥3 underlying medical conditions received at least one vaccine dose, compared with 24.0% of the overall study population, suggesting potential risk-based vaccination, which could lead to underestimation of the vaccine’s effect. In contrast, only 13.5% of individuals with a history of hospitalization within six months before CED received at least one dose, suggesting a possible healthy vaccinee effect, which could lead to overestimation of the vaccine’s effect. These findings indicate that both risk-based vaccination intention and a healthy vaccinee effect were likely to be present in our study. Sixth, our study cohort was restricted to individuals with at least one insurance claim within a defined period before CED (12 months or 6 months). This population may therefore have exhibited a higher prevalence of underlying medical conditions or proportion of healthcare utilization than the general population, potentially limiting the generalizability of our findings. Seventh, although this study included a large population, the number of COVID-19-associated hospitalizations was small, reflecting the generally low risk of severe COVID-19 among children and adolescents. Consequently, the estimated incidence rates and incidence rate ratios were accompanied by wide confidence intervals, and our findings should be interpreted with caution.

## 5. Conclusions

In conclusion, receipt of at least two doses of an ancestral monovalent COVID-19 vaccine was associated with a reduced risk of COVID-19-associated hospitalization among children and adolescents in Japan. These findings provide real-world evidence that pediatric COVID-19 vaccination was associated with a lower risk of severe outcomes. Expanding vaccine uptake among eligible pediatric populations may help reduce the future burden of severe disease.

## Figures and Tables

**Table 3:** Characteristics and clinical outcomes of COVID-19-associated hospitalization among children and adolescents aged 6 months to <18 years, 1 January 2022, to 31 March 2023, Japan.

	COVID-19-Associated Hospitalization, no. (%) (n = 93)
Median age at admission (year, IQR)	8 (3–12)
Age group at admission, no. (%)	
6 months to <5 years	35 (37.6)
5 to <12 years	33 (35.5)
12 to <18 years	25 (26.9)
Female sex, no. (%)	46 (49.5)
Presence of underlying medical condition, no. (%)	
Any	28 (30.1)
Neurologic	11 (11.8)
Cardiovascular	7 (7.5)
Respiratory	1 (1.1)
Renal and urologic	3 (3.2)
Gastrointestinal	2 (2.2)
Hematologic or immunologic	2 (2.2)
Metabolic	8 (8.6)
Other congenital or Genetic defect	3 (3.2)
Malignancy	4 (4.3)
Premature and neonatal	7 (7.5)
Number of underlying medical conditions, no. (%)	
0	65 (69.9)
1 to 2	24 (25.8)
≥3	4 (4.3)
History of hospitalization within six months before CED	16 (17.2)
COVID-19 vaccination status at admission, no. (%)	
Unvaccinated	82 (88.2)
One dose	1 (1.1)
Two doses	8 (8.6)
Three doses	2 (2.2)
Procedure during hospitalization, no. (%)	
Oxygen support without ventilation	4 (4.3)
Mechanical ventilation with intubation	2 (2.2)
Diagnosis during hospitalization, no. (%)	
Vomiting/Diarrhea	21 (22.6)
Hypovolemia	20 (21.5)
Hypoglycemia	5 (5.4)
Seizure	9 (9.7)
In-hospital death, no. (%)	0
Median length of hospitalization period (day, IQR)	4 (3–6)

Abbreviations; COVID-19, coronavirus disease 2019; IQR, interquartile range, CED, cohort entry date.

**Table 4:** Crude incidence rates of COVID-19-associated hospitalization among children and adolescents aged 6 months to <18 years by vaccination status, 1 January 2022, to 31 March 2023, Japan.

	No. of Event	Person-Months	Incidence Rate ^a^ of COVID-19-Associated Hospitalization (95% CI)
**Primary outcome ^b^**			
Unvaccinated	82	1,803,173	4.5 (3.6 to 5.6)
Partially vaccinated	1	30,909	3.2 (0.1 to 18.0)
Fully vaccinated	10	433,502	2.3 (1.1 to 4.2)
**Secondary outcome ^c^**			
Unvaccinated	78	1,803,173	4.3 (3.4 to 5.4)
Partially vaccinated	0	30,909	0 (0 to 11.9)
Fully vaccinated	9	433,502	2.1 (0.9 to 3.9)

^a^: Incidence rate of COVID-19-associated hospitalization per 100,000 person-months. ^b^: Primary outcome was COVID-19-associated hospitalization, defined as any hospital admission with both: (1) an International Classification of Diseases, Tenth Revision (ICD-10) diagnosis codes of U07.1 (COVID-19), B34.2 (coronavirus infection, unspecified), or U10.9 (multisystem inflammatory syndrome in children: MIS-C), and (2) a procedure code indicating COVID-19-associated hospitalization (Supplementary Table S2). ^c^: Secondary outcome was COVID-19-associated hospitalization, excluding hospitalizations with trauma-related diagnoses (ICD-10 codes S00–S99) or surgical procedure codes. Partially vaccinated was defined as receiving one dose of an ancestral monovalent COVID-19 vaccine until <14 days after the second dose. Fully vaccinated was defined as receiving at least two doses, with ≥14 days having elapsed since the second dose. Abbreviations; COVID-19, coronavirus disease 2019; CI, confidence interval.

## Data Availability

The data are not available due to privacy and ethical restrictions.

## Supplementary Materials

The following supporting information can be downloaded at: https://www.mdpi.com/article/10.3390/children13020183/s1.
