# Supplementary material for: Incidence of COVID-19-Associated Hospitalization by Vaccination Status in Children and Adolescents During Omicron-Dominant Period in Japan: The VENUS Study
Source: Children (Basel). 2026 Jan 28;13(2):183. doi: 10.3390/children13020183 (PMC12939590; doi:10.3390/children13020183)
Supplement: Supplementary file 1 [file children-13-00183-s001.zip › children-4088328-supplementary/20260122_supplementary material.pdf]

Table S1. Overview of Japanese health insurance programs administered by municipal governments included in this study

Program name	Target population	Key characteristics
National Health Insurance	Self-employed individuals, retirees, and others not covered by employment-based insurance	Part of Japan’s universal health coverage system; provides standard medical insurance benefits with co-payments
Public Assistance	Individuals with low-income meeting criteria for public assistance	Medical costs are fully covered; no patient co-payment
Children’s Medical Expense Subsidy Program	Children (age eligibility defined by each municipality)	Out-of-pocket payments are reduced or waived; eligibility criteria, coverage, and co-payments vary by municipality
Single-Parent Household Medical Expense Subsidy Program	Children and caregivers in single-parent households	Provides financial support for medical expenses; details vary by municipality
Severely Disabled Persons Medical Expense Subsidy Program	Individuals with certified severe physical, intellectual or mental disabilities	Medical co-payments are reduced or waived depending on disability severity and local policy
Medical System for Services and Supports for Persons with Disabilities	Children with designated chronic and rare diseases	Nationally defined eligibility and disease list; co-payments are capped based on household income

Table S2. List of medical procedure codes for COVID-19-associated hospitalization

**Category II infectious disease code**	
Category II infectious disease	190101870, 190127710, 190136210, 190232670 190237550
**COVID-19-associated codes**	
COVID-19-related hospitalization charges	190232710, 190237610, 190237710
COVID-19-related hospitalization charges for emergency ward	190221450, 190221550, 190221650, 190222610, 190225950, 190226050, 190226150, 190232910, 190234610, 193522610, 193522710, 193522810, 193522910, 193523010, 193523110, 193525710, 193525810, 193525910, 193526010, 193526110, 193528710, 193528810, 193528910, 193529010, 193529110, 193549010, 193549110, 193549210, 193549310, 193551910, 193552010, 193552110, 193552210, 193552310, 193554910, 193555010, 193555110, 193555210, 193555310, 193573910, 193574510, 193580210, 193580810, 193581410, 193575110, 190221750, 190221850, 190221950, 190222710, 190226250, 190226350, 190226450, 190233010, 190234710, 193523210, 193523310, 193523410, 193523510, 193523610, 193526210, 193526310, 193526410, 193526510, 193526610, 193529210, 193529310, 193529410, 193529510, 193529610, 193549410, 193549510, 193549610, 193549710, 193549810, 193552410, 193552510, 193552610, 193552710, 193552810, 193555410, 193555510, 193555610, 193555710, 193555810, 193574010, 193574610, 193575210, 193580310, 193580910, 193581510, 190222050, 190222150, 190222250, 190222810, 190226550, 190226650, 190226750, 190228910, 190229010, 190229110, 190229210, 190229310, 190229410, 190233110, 190234810, 190236310, 190236410, 193523710, 193523810, 193523910, 193524010, 193524110, 193524210, 193524310, 193524410, 193524510, 193524610, 193526710, 193526810, 193526910, 193527010, 193527110, 193527210, 193527310, 193527410, 193527510, 193527610, 193529710, 193529810, 193529910, 193530010, 193530110, 193530210, 193530310, 193530410, 193530510, 193530610, 193549910, 193550010, 193550110, 193550210, 193550310, 193550410, 193550510, 193550610, 193550710, 193550810, 193552910, 193553010, 193553110, 193553210, 193553310, 193553410, 193553510, 193553610,

	193553710, 193553810, 193555910, 193556010, 193556110, 193556210, 193556310, 193556410, 193556510, 193556610, 193556710, 193556810, 193574110, 193574210, 193574710, 193574810, 193575310, 193575410, 193580410, 193580510, 193581010, 193581110, 193581610, 193581710, 190222350, 190222450, 190222550, 190222910, 190226850, 190226950, 190227050, 190229510, 190229610, 190229710, 190229810, 190229910, 190230010, 190230110, 190230210, 190233210, 190234910, 190236510, 190236610, 193524710, 193524810, 193524910, 193525010, 193525110, 193525210, 193525310, 193525410, 193525510, 193525610, 193527710, 193527810, 193527910, 193528010, 193528110, 193528210, 193528310, 193528410, 193528510, 193528610, 193530710, 193530810, 193530910, 193531010, 193531110, 193531210, 193531310, 193531410, 193531510, 193531610, 193550910, 193551010, 193551110, 193551210, 193551310, 193551410, 193551510, 193551610, 193551710, 193551810, 193553910, 193554010, 193554110, 193554210, 193554310, 193554410, 193554510, 193554610, 193554710, 193554810, 193556910, 193557010, 193557110, 193557210, 193557310, 193557410, 193557510, 193557610, 193557710, 193557810
COVID-19-related management charges for high care unit	190224250, 190224350, 190227950, 190228050, 190233710, 190233810, 190235410, 190235510, 193539410, 193539510, 193539610, 193539810, 193539910, 193540010, 193540210, 193540310, 193540410, 193540610, 193540710, 193540810, 193541010, 193541110, 193541210, 193541410, 193541510, 193541610, 193565510, 193565610, 193565710, 193565810, 193565910, 193566010, 193566110, 193566210, 193566310, 193566410, 193566510, 193566610, 193566710, 193566810, 193566910, 193567010, 193567110, 193567210, 193577510, 193577610, 193577710, 193577810, 193577910, 193578010, 193583810, 193583910, 193584010, 193584110, 193584210, 193584310, 190224410, 190224510
COVID-19-related management charges for stroke care unit	190224650, 190228150, 190233910, 190235610, 193541710, 193541810, 193541910, 193542010, 193542110, 193542210, 193542310, 193542410, 193542510, 193567310, 193567410, 193567510, 193567610, 193567710, 193567810, 193567910, 193568010, 193568110, 193578110, 193578210, 193578310,

	193584410, 193584510, 193584610, 190224710
COVID-19-related management charges for pediatric intensive care unit	190224850, 190224950, 190228250, 190228350, 190234010, 190235710, 193542610, 193542710, 193542810, 193542910, 193543010, 193543110, 193543210, 193543310, 193543410, 193543510, 193543610, 193543710, 193568210, 193568310, 193568410, 193568510, 193568610, 193568710, 193568810, 193568910, 193569010, 193569110, 193569210, 193569310, 193578410, 193578510, 193578610, 193584710, 193584810, 193584910, 190225010
COVID-19-related management charges for intensive care unit	190223050, 190223150, 190223250, 190223350, 190223450, 190223550, 190223650, 190223750, 190227150, 190227250, 190227350, 190227450, 190227550, 190227650, 190227750, 190227850, 190230310, 190230410, 190230510, 190230610, 190230710, 190230810, 190230910, 190231010, 190233310, 190233410, 190233510, 190233610, 190235010, 190235110, 190235210, 190235310, 190236710, 190236810, 190236910, 190237010, 193532110, 193532210, 193532310, 193532410, 193532510, 193532610, 193532710, 193532810, 193532910, 193533010, 193533110, 193533210, 193533310, 193533410, 193533510, 193533610, 193533710, 193533810, 193533910, 193534010, 193534110, 193534210, 193534310, 193534410, 193534510, 193534610, 193534710, 193534810, 193534910, 193535010, 193535110, 193535210, 193535310, 193535410, 193535510, 193535610, 193535710, 193535810, 193535910, 193536010, 193536110, 193536210, 193536310, 193536410, 193536510, 193536610, 193536710, 193536810, 193536910, 193537010, 193537110, 193537210, 193537310, 193537410, 193537510, 193537610, 193537710, 193537810, 193537910, 193538010, 193538110, 193538210, 193538310, 193538410, 193538510, 193538610, 193538710, 193558310, 193558410, 193558510, 193558610, 193558710, 193558810, 193558910, 193559010, 193559110, 193559210, 193559310, 193559410, 193559510, 193559610, 1935
COVID-19-related management charges for Neonatal intensive care unit	190225150, 190228450, 190234110, 190235810, 193543810, 193543910, 193544010, 193544410, 193544510, 193544610, 193545010, 193545110, 193545210, 193569410, 193569510, 193569610, 193570010, 193570110, 193570210, 193570610, 193570710, 193570810, 193578710, 193578910, 193579110,

	193585010, 193585210, 193585410, 190225310, 190225250, 190228550, 190234210, 190235910, 193544110, 193544210, 193544310, 193544710, 193544810, 193544910, 193545310, 193545410, 193545510, 193569710, 193569810, 193569910, 193570310, 193570410, 193570510, 193570910, 193571010, 193571110, 193578810, 193579010, 193579210, 193585110, 193585310, 193585510
COVID-19-related management charges for maternal-fetal intensive care unit	190225450, 190228650, 190234310, 190236010, 193545610, 193545710, 193545810, 193546210, 193546310, 193546410, 193546810, 193546910, 193547010, 193571210, 193571310, 193571410, 193571810, 193571910, 193572010, 193572410, 193572510, 193572610, 193579310, 193579510, 193579710, 193585610, 193585810, 193586010, 190225450, 190228650, 190234310, 190236010, 193545610, 193545710, 193545810, 193546210, 193546310, 193546410, 193546810, 193546910, 193547010, 193571210, 193571310, 193571410, 193571810, 193571910, 193572010, 193572410, 193572510, 193572610, 193579310, 193579510, 193579710, 193585610, 193585810, 193586010, 190225550, 190228750, 190234410, 190236110, 193545910, 193546010, 193546110, 193546510, 193546610, 193546710, 193547110, 193547210, 193547310, 193571510, 193571610, 193571710, 193572110, 193572210, 193572310, 193572710, 193572810, 193572910, 193579410, 193579610, 193579810, 193585710, 193585910, 193586110

Abbreviations; COVID-19, coronavirus disease 2019.

Table S3. Japanese version of ICD-10 code list for pediatric complex chronic conditions

Category		Japanese version of the ICD-10 codes
Neurological	Brain and spinal cord malformations	Q00-Q07, G90.1
	Mental retardation	F71-F73
	CNS degeneration and diseases	E75.0, E75.1, E75.2, E75.4, F84.2, G11.1-G11.4, G11.8, G11.9, G12.0-G12.2, G12.8, G12.9, G31, G32, G93.8, G93.9, G94, G91.1, G31.9, G25.3, G95, G90.9, Q85.1
	Infantile cerebral palsy	G80
	Epilepsy	G40, G41
	Other disorders of CNS	G37.1, G37.2, G37.8, G81.9, G82.9, G82.5, G83.5, G83.9, G93.1, G93.5, R40.3
	Occlusion of cerebral arteries	I63.3, I63.5
	Muscular dystrophies and myopathies	G71, G72
	Movement diseases	G10, G20, G21, G23, G24, G25, G80
	Devices	Z98.2
Cardiovascular	Heart and great vessel malformations	Q20, Q21.2-Q24, Q25.1-Q26, Q28.2, Q28.3, Q28.9
	Endocardium diseases	I34.0, I34.8, I36.0, I36.8, I37.0, I37.8
	Cardiomyopathies	I42, I43, I51.5
	Conduction disorder	I44, I45, I47, I48, I49.0
	Dysrhythmias	I49.1-I49.5, I49.8, I49.9, R00.1
	Other	I27.0, I27.1, I27.2, I27.8, I27.9, I50.9, I51.7, I51.8, I63, Z95.1
	Devices	Z95
	Transplantation	Z94.1
Respiratory	Respiratory malformations	Q30-Q34, P280
	Chronic respiratory diseases	I43, J84.1, J96.2, Z90.2
	Cystic fibrosis	E84
	Other	NA
	Devices	J95, Z43.0, Z93.0, Z99.0, J95.8,
	Transplantation	T86.8, Z94.2
Renal and Urologic	Congenital anomalies	Q60-Q64

	Chronic renal failure	N18
	Other	Z90.5, Z90.6,
	Chronic bladder diseases	G83.4, N31.2, N31.9
	Devices	Z93.5, Z93.6, Z99.2, Z43.5, Z43.6, Z46.6
	Transplantation	T86.1, Z940
Gastrointestinal	Congenital anomalies	Q39.0-Q39.4, Q41-Q45
	Chronic liver disease and cirrhosis	K73, K74, K75.4, K760-K763, K765, K768
	Inflammatory bowel diseases	K50, K51
	Other	I82.0, K55.1, K56.2, K59.3, Z98.0, Z90.3, Z90.4
	Devices	K94.2, Z93.1-Z93.4, Z43.1-Z43.4, Z46.5
	Transplantation	T86.4, T86.8, Z94.4, Z94.8
Hematologic or immunologic	Hereditary anemias	D55-D58
	Aplastic anemias	D60-D61, D71
	Hereditary immunodeficiency	D80-D89, D72.0, M30.3, M35.9
	Coagulation/hemorrhagic	D66, D68.2, D69.4
	Leukopenia	D70.0, D70.4
	Hemophagocytic Syndromes	D76.1-D76.3
	Sarcoidosis	D86
	Acquired immunodeficiency	B20-B24
	Polyarteritis nodosa and related conditions	M30.0, M31.0, M31.1, M31.3, M31.4, M31.6
	Diffuse diseases of connective tissue	M32.1, M33.9, M34.0, M34.1, M34.9
	Other	N/A
	Devices	N/A
	Transplantation	N/A
Metabolic	Amino acid metabolism	E70.0, E70.2, E70.3, E70.4, E70.8, E71.0-E71.5, E72.0-E72.4, E72.8, E72.9
	Carbohydrate metabolism	E74.0-E74.4, E74.8, E74.9
	Lipid metabolism	E75, E77.0, E77.1, E78.0-E78.4, E78.5-E78.9, E88.1, E88.8
	Storage disorder	E76.0-E76.3, E85
	Other metabolic disorders	E79.1, E79.8, E80.4-E80.7, E83.0, E83.1, E83.3, E83.4, D84.1, E88, H49.8

	Endocrine disorders	E00.9, E23.0, E23.2, E22.2, E23.3, E23.7, E24.0, E24.2, E24.3, E24.8, E24.9, E26.8, E25.0, E25.8, E25.9,
	Devices	Z46.8, Z96.4
	Transplantation	N/A
Other Congenital or Genetic Defect	Chromosomal anomalies	Q90.9, Q91.3, Q91.4, Q91.7, Q92.8, Q93, Q95.0, Q96.9, Q97, Q98, Q99.8, Q99.9
	Bone and join anomalies	E34.3, M41.0, M41.2, M41.3, M41.8, M41.9, M43.3 M96.5, Q72.2, Q75.0, Q75.2, Q75.9, Q76.0-Q76.2, Q76.4-Q76.7, Q77, Q78.0-Q78.4, Q78.8, Q78.9
	Diaphragm and abdominal wall	K44.9, Q79.0-Q79.5, Q79.9
	Other congenital anomalies	Q81, Q87.1-Q87.3, Q87.4, Q87.8, Q89.7, Q89.9, Q99.2
Malignancy	Neoplasms	C00-C96, D01-D09, D3A.0, D37-D49, Q85.0
	Devices	N/A
	Transplantation	T86.0, Z94.8
Premature and Neonatal	Fetal malnutrition	P05
	Extreme immaturity	P07
	Cerebral hemorrhage at birth	P10.0, P10.1, P10.4, P52.4, P52.8
	Spinal cord injury at birth	P11.5
	Birth asphyxia	P21.0, P21.9, P84
	Respiratory diseases	P25.0-P25.3, P25.8, P27.0, P27.1, P27.8
	Hypoxic-ischemic encephalopathy	P91.6
	Other	P35.0, P35.1, P25.2, P56.0, P57.0, P57.8, P61.3, P61.4, P77.3, P83.2, P91.2
Miscellaneous, Not Elsewhere Classified	Devices	T84, T86, T87, Y83.1, Y83.3, Z99.8
	Transplantation	T86.5, T86.9, T86.8

Codes modified based on a reference (Feudtner C, et al. Pediatric complex chronic conditions classification system version 2: updated for ICD-10 and complex medical technology dependence and transplantation. BMC Pediatr. 2014;14:199).

Table S4. Characteristics and clinical outcomes of COVID-19-associated hospitalization excluding hospitalizations with trauma-related diagnoses or surgical procedure codes among children and adolescents aged 6 months to <18 years, January 1, 2022, to March 31, 2023, Japan.

	COVID-19-associated hospitalizations excluding hospitalizations with trauma-related diagnoses or surgical procedure codes*, no. (%) (n=87)
Median age at admission (year, IQR)	8 (3–12)
Age group at admission, no. (%)	
6 months to <5 years	32 (36.8)
5 to <12 years	31 (35.6)
12 to <18 years	24 (27.6)
Female sex, no. (%)	43 (49.4)
Presence of underlying medical condition, no. (%)	
Any	27 (30.1)
Neurologic	11 (12.6)
Cardiovascular	7 (8.0)
Respiratory	1 (1.1)
Renal and urologic	3 (3.4)
Gastrointestinal	2 (2.3)
Hematologic or immunologic	2 (2.3)
Metabolic	8 (9.2)
Other congenital or Genetic defect	3 (3.4)
Malignancy	3 (3.4)
Premature and neonatal	7 (8.0)
Number of underlying medical conditions, no. (%)	
0	60 (69.0)
1 to 2	23 (26.4)
≥3	4 (4.6)
History of hospitalization within six months before CED	16 (18.4)
COVID-19 vaccination status at admission, no. (%)	
Unvaccinated	78 (89.7)
One dose	0
Two doses	8 (9.2)
Three doses	1 (1.1)
Procedure during hospitalization, no. (%)	

Oxygen support without ventilation	2 (2.3)
Mechanical ventilation with intubation	2 (2.3)
Diagnosis during hospitalization, no. (%)	
Vomiting/Diarrhea	20 (23.0)
Hypovolemia	20 (23.0)
Hypoglycemia	5 (5.7)
Seizure	9 (10.3)
In-hospital death, no. (%)	0
Median length of hospitalization period (day, IQR)	4 (3–6)

Abbreviations; COVID-19, coronavirus disease 2019; IQR, interquartile range; CED, cohort entry date.

*This definition was defined as COVID-19-associated hospitalizations excluding trauma-related diagnoses (ICD-10 codes: S00–S99) or surgical procedure codes.

Table S5. Crude incidence rates of COVID-19-associated hospitalization stratified by subgroups among children and adolescents aged 6 months to <18 years by vaccination status, January 1, 2022, to March 31, 2023, Japan.

	No. of event	Person-months	Incidence rate[a] of COVID-19-associated hospitalization (95% CI)
**Age group**			
6 months to <5 years			
Unvaccinated	35	578,931	6.0 (4.2 to 8.4)
Partially vaccinated	0	1,503	0 (0 to 245.4)
Fully vaccinated	0	1,954	0 (0 to 188.8)
5 to <12 years			
Unvaccinated	32	976,714	3.3 (2.2 to 4.6)
Partially vaccinated	0	19,917	0 (0 to 18.5)
Fully vaccinated	1	105,838	0.9 (0.02 to 5.3)
12 to <18 years			
Unvaccinated	15	247,527	6.1 (3.4 to 10.0)
Partially vaccinated	1	9,489	10.5 (0.3 to 58.7)
Fully vaccinated	9	325,710	2.8 (1.3 to 5.2)
**Presence of underlying medical condition**			
With underlying medical condition			
Unvaccinated	28	117,762	23.8 (15.8 to 34.4)
Partially vaccinated	0	2,125	0 (0 to 173.6)
Fully vaccinated	0	35,239	0 (0 to 10.5)
Without underlying medical condition			
Unvaccinated	54	1,685,411	3.2 (2.4 to 4.2)
Partially vaccinated	1	28,784	3.5 (0.1 to 19.4)
Fully vaccinated	10	398,263	2.5 (1.2 to 4.6)
**Period of dominant Omicron subvariants**			
BA.1/BA.2 (January–June 2022)			
Unvaccinated	42	742,352	5.7 (4.1 to 7.6)
Partially vaccinated	1	17,677	5.7 (0.1 to 31.5)
Fully vaccinated	4	139,150	2.9 (0.8 to 7.4)
BA.5 (July–November 2022)			
Unvaccinated	30	595,470	5.0 (3.4 to 7.2)
Partially vaccinated	0	7,904	0 (0 to 46.7)
Fully vaccinated	4	161,692	2.5 (0.7 to 6.3)
BA.5/BQ.1 (December 2022–March 2023)			
Unvaccinated	10	465,351	2.1 (1.0 to 4.0)

Partially vaccinated	0	5,328	0 (0 to 69.2)
Fully vaccinated	2	132,660	1.5 (0.2 to 5.4)

a: Incidence rate of COVID-19-associated hospitalization per 100,000 person-months.

Partially vaccinated was defined as receiving one dose of an ancestral monovalent COVID-19 vaccine until <14 days after the second dose.

Fully vaccinated was defined as receiving at least two doses, with ≥14 days having elapsed since the second dose.

Abbreviations; COVID-19, coronavirus disease 2019; CI, confidence interval.

Table S6. Crude incidence rates of COVID-19-associated hospitalization excluding hospitalizations with trauma-related diagnoses or surgical procedure codes stratified by subgroups among children and adolescents aged 6 months to <18 years by vaccination status, January 1, 2022, to March 31, 2023, Japan.

	No. of event	Person-months	Incidence rate[a] of COVID-19-associated hospitalization excluding hospitalizations with trauma-related diagnoses or surgical procedure codes* (95% CI)
**Age group**			
6 months to <5 years			
Unvaccinated	32	578,931	5.5 (3.8 to 7.8)
Partially vaccinated	0	1,503	0 (0 to 245.4)
Fully vaccinated	0	1,954	0 (0 to 188.8)
5 to <12 years			
Unvaccinated	31	976,714	3.2 (2.2 to 4.5)
Partially vaccinated	0	19,917	0 (0 to 18.5)
Fully vaccinated	0	105,838	0 (0 to 3.5)
12 to <18 years			
Unvaccinated	15	247,527	6.1 (3.4 to 10.0)
Partially vaccinated	0	9,489	0 (0 to 38.9)
Fully vaccinated	9	325,710	2.8 (1.3 to 5.2)
**Presence of underlying medical condition**			
With underlying medical condition			
Unvaccinated	27	117,762	22.9 (15.1 to 33.4)
Partially vaccinated	0	2,125	0 (0 to 173.6)
Fully vaccinated	0	35,239	0 (0 to 10.5)
Without underlying medical condition			
Unvaccinated	51	1,685,411	3.0 (2.3 to 4.0)
Partially vaccinated	0	28,784	0 (0 to 12.8)
Fully vaccinated	9	398,263	2.3 (1.0 to 4.3)
**Period of dominant Omicron subvariants**			
BA.1/BA.2 (January–June 2022)			
Unvaccinated	39	742,352	5.3 (3.7 to 7.2)
Partially vaccinated	0	17,677	0 (0 to 20.9)
Fully vaccinated	4	139,150	2.9 (0.8 to 7.4)
BA.5 (July–November 2022)			
Unvaccinated	30	595,470	5.0 (3.4 to 7.2)
Partially vaccinated	0	7,904	0 (0 to 46.7)

Fully vaccinated	4	161,692	2.5 (0.7 to 6.3)
BA.5/BQ.1 (December 2022–March 2023)			
Unvaccinated	9	465,351	1.9 (0.9 to 3.7)
Partially vaccinated	0	5,328	0 (0 to 69.2)
Fully vaccinated	1	132,660	0.8 (0 to 4.2)

a: Incidence rate of COVID-19-associated hospitalization per 100,000 person-months.

Partially vaccinated was defined as receiving one dose of an ancestral monovalent COVID-19 vaccine until <14 days after the second dose.

Fully vaccinated was defined as receiving at least two doses, with ≥14 days having elapsed since the second dose.

*This definition was defined as COVID-19-associated hospitalizations excluding trauma-related diagnoses (ICD-10 codes: S00–S99) or surgical procedure codes.
